# Relationship between Preoperative Lower Back Pain and Severe Postoperative Pain after Gynecologic Laparoscopy: A Prospective Observational Study

**DOI:** 10.3390/jcm11154295

**Published:** 2022-07-24

**Authors:** Jae-Yen Song, Minsuk Chae, Hyunjoon Lee, Young-Eun Moon

**Affiliations:** 1 Department of Obstetrics and Gynecology, Seoul St. Mary’s Hospital, College of Medicine, The Catholic University of Korea, Seoul 06591, Korea; jaeyen77@catholic.ac.kr; 2 Department of Anesthesiology and Pain Medicine, Seoul St. Mary’s Hospital, College of Medicine, The Catholic University of Korea, Seoul 06591, Korea; shscms@catholic.ac.kr (M.C.); fhdvkfguswns@naver.com (H.L.)

**Keywords:** gynecologic surgery, laparoscopy, lower back pain, postoperative pain

## Abstract

We hypothesized that preoperative lower back pain (LBP) may be associated with the severity of postoperative pain after gynecologic laparoscopy. This prospective observational study aimed to investigate the association between preoperative LBP and postoperative pain. We assessed the intensity of LBP before surgery and the postoperative pain after surgery. The abilities of preoperative LBP intensity, age, body mass index, and anesthetic duration time to predict moderate-to-severe postoperative pain were measured using receiver operating characteristic analysis. The data of 148 patients were analyzed. Only preoperative LBP intensity showed a significant association with moderate-to-severe postoperative pain (area under the curve, 0.71; 95% confidence interval, 0.63–0.79; *p* < 0.001). Preoperative LBP rated three on a numeric rating scale (NRS) had the highest combined sensitivity (75.3%) and specificity (58.3%). Patients with LBP above NRS 3 had more severe postoperative pain than those who did not (pain score 5.3 ± 2.2 vs. 3.9 ± 1.9, *p* < 0.001), leading to more opioid requirement in the recovery room (48.5% vs. 27.5%, *p* = 0.014). Preoperative LBP intensity is a useful factor for identifying patients at risk for pain after gynecologic laparoscopy.

## 1. Introduction

Although laparoscopic surgery has advantages such as enhanced postoperative recovery and decreased hospital stay in comparison with open surgery [1,2], the incidence of moderate-to-severe acute postoperative pain, especially in the recovery room, remains up to 70% [3,4]. Moreover, pain intensities vary even after the same surgery, which suggests the need for individual evaluation of risk factors for postoperative pain [5,6]. Despite recent advances in anesthesiology, personalized preemptive analgesia remains difficult, with overdosing leading to slow recovery and underdosing leading to severe pain [7]. Therefore, it will be valuable to identify patients at a high risk of postoperative pain.

The lithotomy position required during gynecologic laparoscopy is ergonomically difficult on the lower back. Therefore, if the patient has preoperative lower back pain (LBP), the severity of LBP will increase due to the lithotomy position, which naturally leads to a negative impact on postoperative pain. However, to our knowledge, no reports have validated such claims. We hypothesized that preoperative LBP is associated with more severe postoperative pain in the recovery room.

This study aimed to investigate the association between preoperative LBP and postoperative pain after gynecologic laparoscopy and the ability of preoperative LBP intensity to predict moderate-to-severe postoperative pain in the recovery room.

## 2. Materials and Methods

This prospective clinical observational study was conducted according to the guidelines of the Declaration of Helsinki, and approved by the Institutional Review Board of Seoul St. Mary’s Hospital, Catholic University of Korea (approval number: KC20MNSI0130) on 7 March 2021, and registered at ClinicalTrials.gov (NCT04793191). Written informed consent was obtained from 150 female patients scheduled for gynecologic laparoscopy including hysterectomy, adnexectomy, cystectomy or cyst enucleation, and myomectomy.

The exclusion criteria were incapacity to provide consent, emergency surgery, cancer surgery, chronic pain except LBP, chronic substance use, history of psychiatric disease, pregnancy, or lactation. Before starting the study, patients were instructed on how to assess their pain intensity using a numeric rating scale (NRS; 0, no pain; 10, the worst pain imaginable). Moreover, the patients were asked the following question: “Have you had any pain in your lower back? If any, how much is the worst pain you have experienced?” LBP intensity was recorded using the NRS.

All patients underwent standard induction of anesthesia (propofol, remifentanil, and rocuronium) and orotracheal intubation. Anesthesia was maintained with 4–6% desflurane (expired concentration) in 40% air/oxygen (total flow, 4 L/min) to maintain a bispectral index between 30 and 60. Ventilation was controlled mechanically and adjusted to maintain an end-tidal carbon dioxide value between 25 and 40 mmHg throughout the surgery. Additional rocuronium was administered, as required. Laparoscopy was performed under video guidance, with three punctures in the abdomen. The intraperitoneal pressure was maintained at approximately 12 mmHg. To prevent postoperative nausea and vomiting (PONV), all patients received 5 mg intravenous dexamethasone at the beginning of surgery and 75 μg intravenous palonosetron at the end of surgery. For postoperative pain control, multimodal analgesia, consisting of intravenous acetaminophen (500 mg) and ketorolac (30 mg), was administered 30 min before the end of surgery. These non-opioid analgesics were continued throughout the hospital stay in the ward.

After confirming self-respiration, patients were extubated and transferred to the recovery room. Postoperative pain intensity was assessed using the NRS upon arrival in the recovery room and every 15 min thereafter. If the patient complained of pain (NRS > 4), 0.5–1 μg/kg intravenous fentanyl was administered immediately after assessing and recording the pain intensity. If the patient complained of nausea and/or vomiting, 10 mg intravenous metoclopramide was administered. After leaving the recovery room, intravenous patient-controlled analgesia (PCA; fentanyl 15 μg/kg in normal saline 100 mL; basal rate 0 mL/h; bolus 1 mL; lock-out time 10 min) was applied to all patients. In the recovery room, pain intensity, requirement for pain killers, and incidence of PONV were assessed. These were again evaluated in the ward 24 h after surgery.

### Statistical Analysis

The calculation of the sample size was based on the aim of detecting any clinically meaningful value of preoperative LBP intensity to predict moderate-to-severe acute pain (NRS 4–10) in the recovery room, defined as an area under the curve (AUC) of the receiver operating characteristic (ROC) curve of at least 0.7. To detect such differences, 50 patients in each group (no to mild pain, NRS 0–3; moderate to severe pain, NRS 4–10) were deemed necessary. Based on our clinical experience and previous research [7], this event rate between the two groups was unlikely to be 1:1 but more likely 2:1. Consequently, 150 patients were included in this study.

Data were tested for normal distribution using the Kolmogorov–Smirnov test and are presented as means with standard deviations or medians with 25th and 75th percentiles. The independent *t*-test or Mann–Whitney U test was used to analyze quantitative variables, and the chi-squared or Fisher’s exact test were used to analyze qualitative variables. The abilities of preoperative LBP intensity, age, body mass index (BMI), and anesthetic duration to predict moderate-to-severe acute pain (NRS 4–10) were measured using ROC analysis. Cut-off values used for the calculation of sensitivity and specificity were calculated based on goodness of fit (highest combined sensitivity and specificity). Kaplan–Meier curves were used to evaluate the time to rescue fentanyl in the recovery room, using the log-rank test. All analyses were performed using SPSS version 20 (IBM, Armonk, NY, USA). Statistical significance was set at *p* < 0.05.

## 3. Results

Between March 2021 and January 2022, a total of 150 patients meeting the inclusion criteria were included in this study, and two patients were excluded because of conversion to open surgery. Data from 148 patients were analyzed.

**Figure 1:**
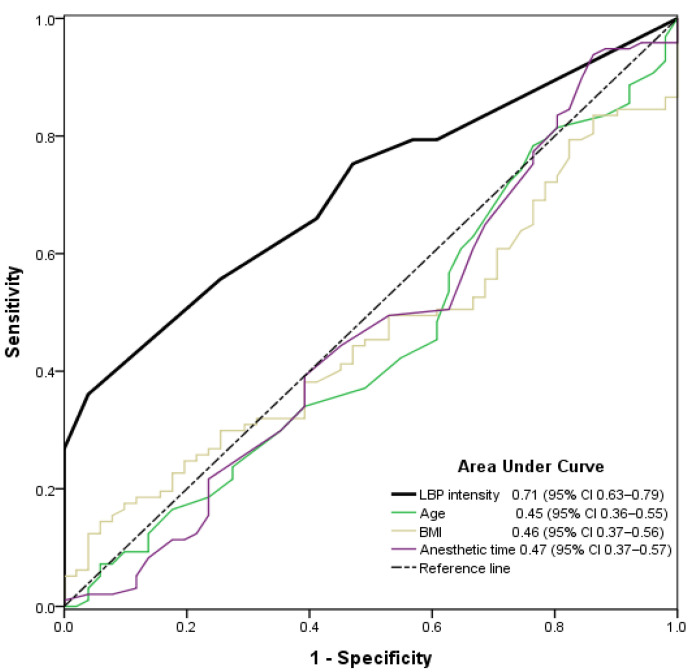
Receiver operating characteristics of preoperative lower back pain intensity, age, body mass index, and anesthetic time to predict moderate-to-severe postoperative pain (numeric rating scale 4–10) in the recovery room. LBP, lower back pain; BMI, body mass index; CI, confidence interval.

There were four pain levels recorded in the recovery room. Patients reported their pain scores as no (NRS 0; *n* = 2), mild (NRS 1–3; *n* = 49), moderate (NRS 4–5; *n* = 41), or severe pain (NRS 6–10; *n* = 56). To investigate the ability to predict moderate-to-severe (NRS 4–10) postoperative pain, ROC analysis was performed for preoperative LBP intensity, age, BMI, and anesthetic duration. Among these parameters, only preoperative LBP intensity showed a significant association with moderate-to-severe pain (AUC 0.71; 95% confidence interval, 0.63–0.79; *p* < 0.001, Figure 1).

Post hoc analysis was performed for the highest combined sensitivity and specificity to distinguish between patients with and without moderate-to-severe postoperative pain in the recovery room. Preoperative LBP at NRS 3 had the highest combined sensitivity (75.3%) and specificity (58.3%). Additionally, we performed binary logistic regression to analyze the factors influencing moderate-to-severe postoperative pain. The presence of the moderate-to-severe postoperative pain (yes or no) was the independent variable. The dependent variables were age, BMI, anesthetic duration, adhesion (yes/no), type of laparoscopic surgery (hysterectomy/adnexectomy/cystectomy or cyst enucleation/myomectomy), and preoperative LBP intensity (NRS ≥ 3/NRS < 3). For the goodness-of-fit test of the multivariate model, we performed the Hosmer–Lemeshow test. We found that patients with preoperative LBP ≥ 3 had a higher risk of moderate-to-severe postoperative pain than the other patients [odds ratio (OR) 3.79, 95% CI 1.77–8.10, *p* = 0.001]. The other factors had no significant association with moderate-to-severe postoperative pain (Table 1).

Consequently, we compared patients who had preoperative LBP intensity levels ≥ 3 with those who did not. There were no significant differences in the demographic or surgical data between patients who had preoperative LBP intensity ≥ 3 and those who did not, except preoperative LBP intensity (Table 2).

However, the postoperative pain score in the recovery room was significantly higher in those with LBP intensity ≥ 3 than in those with LBP intensity < 3 (5.3 ± 2.2 vs. 3.9 ± 1.9, *p* < 0.001, Table 3). This led to more opioid requirement in the recovery room in the LBP intensity ≥ 3 group (48.5% vs. 27.5%, *p* = 0.014) and more severe postoperative pain scores among patients in the ward (3.9 ± 1.8 vs. 2.7 ± 1.8, *p* = 0.001).

**Figure 2:**
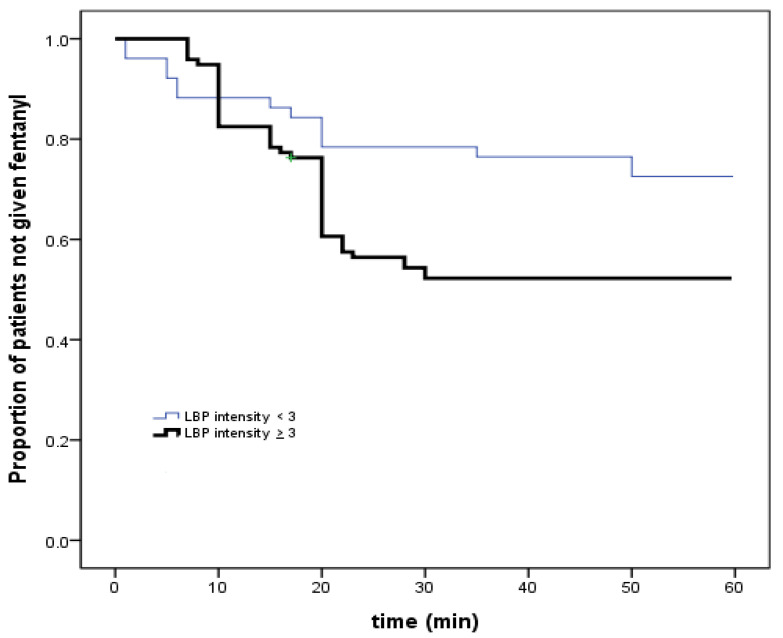
Opioid requirement in patients dichotomized according to preoperative lower back pain intensity. LBP, lower back pain.

Moreover, the proportion of patients who did not receive postoperative fentanyl within 60 min after awakening was significantly lower in those reporting preoperative LBP intensity levels ≥ 3 than in those reporting levels < 3. Postoperative fentanyl was administered significantly earlier in patients with preoperative LBP intensity ≥ 3 than in those with preoperative LBP intensity <3 (Figure 2; *p* = 0.023).

## 4. Discussion

To the best of our knowledge, this is the first study to show that preoperative LBP intensity (≥NRS 3) can be used to predict moderate-to-severe postoperative pain after gynecologic laparoscopy. Preoperative LBP intensity ≥NRS 3 was associated with earlier and more frequent administration of opioids in the recovery room.

Laparoscopy is generally expected to result in less postoperative pain than laparotomy is. However, patients frequently experience more severe pain than expected after awakening from general anesthesia, which was confirmed by our results showing that 66% (41 + 56/148) of patients had moderate-to-severe postoperative pain in the recovery room. This is in accordance with a previous report where laparoscopy caused unexpectedly high levels of postoperative pain, and 70% of the patients did not receive adequate analgesia [3]. These findings strongly imply the need for preoperative identification of patients at a high risk for postoperative pain, which will enable medical staff to provide appropriate individualized analgesic management for these patients.

The well-known risk factors are sex (female vs. male), age (young vs. old), and type of surgery (open surgery vs. laparoscopy) [6,8,9,10]. However, as shown in our study, age failed to predict moderate-to-severe postoperative pain. In addition, although all patients underwent laparoscopic surgery, various levels of pain (none, 1%; mild, 33%; moderate, 28%; severe, 38%) were reported in our study. If so, what might be the potential risk factor for predicting substantial postoperative pain after gynecologic laparoscopy? To investigate this, we focused on the lithotomy position required for gynecologic laparoscopy and hypothesized that this position, anatomically, exerts a negative impact on the lower back, which might be more severe in patients who already have LBP before surgery. Consequently, this hypothesis was confirmed in this study. Given the association of pain with neurogenic inflammation [11,12], we speculate that more severe back pain provokes prostaglandin release, which causes the sensitization of peripheral nociceptors in a surgical setting, leading to more severe postoperative pain. With a preoperative assessment of LBP intensity, approximately 66% (moderate, 28%; severe, 38%) of patients in need of more analgesic management could be identified.

In general, females have more postoperative pain than males [10]. Considering this, our results that show the ability to identify patients with high-risk factors for postoperative pain are meaningful in this clinical setting. It is not difficult to preoperatively assess the severity of LBP. No specific resources, time, or equipment are required. Recognition of patients with LBP above NRS 3 by the medical staff and the application of appropriate management approaches, such as applying less steep head-down tilting during laparoscopy, pad at the lower back, or preemptive and aggressive non-steroidal anti-inflammatory drugs to those patients might effectively decrease the incidence of moderate-to-severe pain; this would also result in a subsequent decrease in the requirement of opioids in the recovery room, leading to a decrease in opioid-related side effects. Further research with a larger sample size is required to validate these hypotheses.

Although opioids are potent analgesics without ceiling effects [13], they are associated with side effects such as sedation, respiratory depression, and PONV [14,15,16]. PONV is more common in females and in laparoscopic surgery. We expected that patients with preoperative LBP intensity < 3 would require fewer opioids; consequently leading to a decrease in PONV. However, although the incidence of PONV is lower in patients with preoperative LBP intensity < 3 than in those with LBP intensity ≥ 3, these differences failed to achieve statistical significance. There are several possible reasons for this finding. First, we aggressively provided prophylactic antiemetics with dexamethasone and palonosetron during surgery. This combination of antiemetics is known to be very effective and mandatory for the prevention of PONV [17]. Second, the sample size of our study may have been too small to detect a significant effect on the incidence of PONV.

This study has some limitations. First, this finding might not extend to other surgeries, such as laparotomy. However, considering the overwhelmingly large proportion of laparoscopies performed for gynecologic benign disease [2], our results are meaningful and clinically relevant. Second, this study evaluated pain intensity for only 1 day postoperatively, which may not reflect chronic pain after postsurgical recovery and wound healing. To validate this, a more sophisticated study is required. Third, we did not study pain on classification (somatic, visceral, or psychogenic). It would have been more ideal to consider this classification. However, given that these factors of pain have complex interactions with each other, the study according to this classification should be done in the future research based on a more sophisticated design.

In conclusion, this study shows that preoperative LBP intensity above NRS 3 is associated with more severe postoperative pain and with earlier and more frequent administration of opioids after gynecologic laparoscopy. Preoperative LBP intensity is a useful factor for identifying patients at risk for pain after surgery, which can lead to more appropriate management of these patients. In addition, a preoperative assessment of LBP can be easily performed without specific equipment or training.

## Figures and Tables

**Table 1:** Binary logistic regression to evaluate the effect of variables on the probability of moderate-to-severe pain in the recovery room.

	OR	95% CI	*p* Value
Age (unit: year)	1.00	0.96–1.04	0.974
BMI (unit: kg/m^2^)	1.00	0.89–1.12	0.977
Anesthetic duration (unit: min)	1.00	0.99–1.01	0.774
Adhesion (Yes/No)	0.79	0.36–1.76	0.565
Surgery type (reference: myomectomy)			0.312
Hysterectomy	0.68	0.17–2.74	0.585
Adnexectomy	1.12	0.16–7.63	0.912
Cystectomy or cyst enucleation	1.77	0.43–7.24	0.427
Preoperative LBP intensity (NRS ≥ 3/NRS < 3)	3.79	1.77–8.10	0.001
Nagelkerke’s R^2^ =0.15, Hosmer & Lemeshow’s χ^2^ = 8.863, *p* = 0.354.			

OR, odds ratio; CI, confidence interval; BMI, body mass index; LBP, lower back pain; NRS, numeric rating scale.

**Table 2:** Demographic and surgical data in patients dichotomized for preoperative lower back pain intensity. Values are as means ± SD or number (%).

	LBP ≥ 3 (*n* = 97)	LBP < 3 (*n* = 51)	*p* Value
*Preoperative*			
Age; year	41.7 ± 9.5	42.8 ± 11.5	0.538
BMI; kg/m^2^	23.0 ± 3.9	22.6 ± 2.6	0.489
LBP intensity (NRS)	5.3 ± 1.5	0.4 ± 0.8	<0.001
*Surgical*			
Duration of anesthetic time (min)	142.9 ± 45.3	139.6 ± 37.5	0.654
Intraoperative remifentanil use (ug/kg/min)	0.10 ± 0.04	0.10 ± 0.03	0.670
Type of surgery			
Hysterectomy ^a^	54 (55.7)	23 (45.1)	0.493
Myomectomy ^b^	9 (9.3)	5 (9.8)	
Cystectomy/cyst enucleation only	28 (28.9)	17 (33.3)	
Adnexectomy only	6 (6.1)	6 (11.8)	

LBP, lower back pain; BMI, body mass index; NRS, numeric rating scale. ^a^ With or without adnexectomy ^b^ With or without cystectomy/cyst enucleation

**Table 3:** Postoperative data in patients dichotomized for preoperative lower back pain intensity. Values are as means ± SD or number (%).

	LBP ≥ 3 (*n* = 97)	LBP < 3 (*n* = 51)	*p* Value
*0.5 h after surgery*			
Pain, NRS (0–10)	5.3 ± 2.2	3.9 ± 1.9	<0.001
Opioid requirement	47 (48.5)	14 (27.5)	0.014
PONV incidence	11 (11.3)	4 (7.8)	0.503
*24 h after surgery*			
Pain, NRS (0–10)	3.9 ± 1.8	2.7 ± 1.8	0.001
Opioid requirement	36 (37.1)	15 (29.4)	0.349
PONV incidence	37 (38.1)	12 (23.5)	0.073

LBP, lower back pain; NRS, numeric rating scale; PONV, postoperative nausea and vomiting.

## Data Availability

The data generated in this study can be shared after a reasonable request to the corresponding author.
